# A Physics-Inspired Mechanistic Model of Migratory Movement Patterns in Birds

**DOI:** 10.1038/s41598-017-09270-6

**Published:** 2017-08-29

**Authors:** Christopher Revell, Marius Somveille

**Affiliations:** 1 Cavendish Laboratory, Department of Physics, University of Cambridge, Cambridge, United Kingdom; 2 Edward Grey Institute, Department of Zoology, University of Oxford, Oxford, United Kingdom

## Abstract

In this paper, we introduce a mechanistic model of migratory movement patterns in birds, inspired by ideas and methods from physics. Previous studies have shed light on the factors influencing bird migration but have mainly relied on statistical correlative analysis of tracking data. Our novel method offers a bottom up explanation of population-level migratory movement patterns. It differs from previous mechanistic models of animal migration and enables predictions of pathways and destinations from a given starting location. We define an environmental potential landscape from environmental data and simulate bird movement within this landscape based on simple decision rules drawn from statistical mechanics. We explore the capacity of the model by qualitatively comparing simulation results to the non-breeding migration patterns of a seabird species, the Black-browed Albatross (*Thalassarche melanophris*). This minimal, two-parameter model was able to capture remarkably well the previously documented migration patterns of the Black-browed Albatross, with the best combination of parameter values conserved across multiple geographically separate populations. Our physics-inspired mechanistic model could be applied to other bird and highly-mobile species, improving our understanding of the relative importance of various factors driving migration and making predictions that could be useful for conservation.

## Introduction

Animal migration, the seasonal movements of billions of organisms across the globe, is one of the most remarkable phenomena in nature. Birds, in particular, can make remarkable journeys between their breeding and non-breeding areas, and their long-distance migrations have been the focus of much research. Previous studies have used statistical analyses of tracking data to infer how migration patterns are influenced by environmental variables^1, 2^, and several factors have been considered to affect the movement patterns and geographical distribution of migratory birds. Among them, two environmental factors in particular have been suggested as major determinants of long-distance movements. First, it has been shown that highly-mobile animals move to track food resources over long distances, targeting seasonally productive areas^3–5^. The spatio-temporal variation in resource density has been found to affect the geographical distribution of birds, particularly during their non-breeding season when birds are no longer constrained by the need to feed their chicks^1, 6, 7^. The second key process likely to affect the movement patterns of birds is wind and its impact on the energetic cost of movement. Birds often use wind for efficient locomotion, or are constrained by it, and wind patterns have been shown to greatly influence the migratory movements of birds^8–12^. However, the statistical approaches used in previous studies are simply correlative, and are insufficient for causal understanding of the underlying forces driving bird migration, and the relative importance of those forces. Without such causal understanding, it is not possible to make reliable predictions for populations that have not yet been tracked, or for future movement patterns under environmental change.

In recent years, there has been significant interest in inter-disciplinary research, particularly between the physical and biological sciences. Such efforts have led to new questions, new solutions, and new ways of thinking for both physicists and biologists^13, 14^, and are helping to transform biology into a numerical, predictive science. Continuing in this tradition, we draw upon ideas and methods from physics to propose a spatially-explicit mechanistic model of migratory movement patterns. Our model aims to explore the underlying forces driving migration from first principles, and in this paperit is specifically adapted to study the long-distance migration of birds. This method integrates the influence of the physical environment into movement decisions made by the birds to track resources, modelling individual movements and predicting population-level distribution patterns. Our approach adds to the previous literature on mechanistic models of large-scale animal movement, from the pioneering work of Kendall^15^, to modelling movement using Levy processes^16^, to models based on movement fields^17^. Our modelling approach shares similarities with previous individual-based models (IBMs) as it simulates individual movements based on simple decision rules, and follows their trajectories over time and space^18^. However, IBMs are typically parameter-rich^19^, while our model has only one biologically-relevant parameter (and another associated with the level of stochasticity). Also, many previous IBMs of bird migration have been designed to describe specific study systems^19–21^, contrasting with the generalised model that we have developed. In addition, our method improves upon physical transport models, which focus on the dynamics of the physical environment but neglect the organism’s movement abilities^18^. Our method also contrasts with dynamic optimisation models, in which individuals move between two points along the path that optimises fitness, but cannot choose their ultimate destinations^18^; our method will predict an unknown destination from a given starting position. Furthermore, our model joins previous work on stochastic dynamic models incorporating fields of energy expenditure, within which individuals adapt their movement strategies to minimise the energy expenditure required by their migratory path^22^. Our model, however, contrasts with the latter as is does not explicitly incorporate energetic expenditure but rather a more abstract definition of an environmental potential field, and also includes attraction at a distance to high productivity areas, reflecting a process of memory or genetic pre-determination.

After describing our new method and its applications, we demonstrate its promise by exploring its ability to predict non-breeding migration patterns from breeding colonies of black-browed albatrosses (*Thalassarche melanophris*). Seabirds, with their exceptional flying abilities, form one of the most highly mobile groups of organisms. Outside of the breeding season, when species nest colonially on land, they roam the world’s oceans and seas with few constraints. Their remarkable long-distance migratory movements are increasingly revealed by tracking data^6, 23–25^. A wide diversity of non-breeding movement patterns have been documented both within and between species, from trans-equatorial^6, 24, 26^, to circumpolar^27^, to complex mixtures of short and intermediate distance migrations^25, 28^. Much recent research has focused on what drives this diversity of non-breeding movement patterns, but has predominantly relied on statistical analysis of tracking data to infer the underlying behavioural and ecological processes^4, 7, 10, 26, 29^. The free movement of non-breeding migration within a relatively open environment for which both environmental and tracking data are readily available makes non-breeding migration of seabirds an excellent system with which to explore the results of our model. The black-browed albatross in particular is an ideal species, since it is the most abundant albatross species of the southern Hemisphere and one of the most mobile and wide-ranging marine predators. The species has a circumpolar distribution, breeding colonially on many geographically separated subantarctic islands and archipelagos, and exhibiting a mix of short and long distance migratory movements during the non-breeding season^30^. This species feeds on a wide variety of prey and mostly targets neritic, productive upwellings and shelf areas^31, 32^. Information on the distributions of black-browed albatrosses outside of the breeding season has been documented for the following populations, each corresponding to a separate breeding colony.

- Islas Diego Ramirez (−56.5°, −68.7°), whose population has been observed to move up the West coast of Chile^23^.
- The Falkland Islands (−51.0°, −61.1°), whose population spends the non-breeding season on the Patagonian Shelf not far from the breeding colony^23, 31^.
- Iles Kerguelen (−49.4°, 70.0°), whose population migrates to the southern coast of Australia, with some moving around Australia into the Tasman Sea and a smaller population instead finding their way to the Benguela Upwelling off South Africa^33, 34^.
- Macquarie Island (−54.5°, 158.9°), whose birds remain around New Zealand and the Tasman Sea for the non-breeding season^35^.
- South Georgia (−54.2°, −36.5°), whose population splits into a set that migrates to the Benguela Upwelling off South Africa, a slightly smaller set that migrates to the Patagonian Shelf off the coast of Argentina, and a few individuals migrating to the southern coast of Australia^36^.

Here, we integrate data for wind and chlorophyll concentration – a proxy for the availability of food – into an environmental potential landscape, and use our mechanistic model to simulate the non-breeding migration patterns within this landscape. We produced exploratory results for populations from the five major breeding colonies discussed above, and made predictions for untracked populations from Campbell Island, the Crozet Islands, and Islas Diego de Almagro.

## Model Description

In our model, we envisaged a bird as a particle moving within a potential landscape, like a ball rolling across a curved surface (a gravitational potential landscape). This potential landscape is defined by environmental data on a two-dimensional 720 × 1440 lattice covering the entire Earth’s surface. The potential at each lattice point is the sum of components derived directly from the factors that are likely to affect the migration behaviour of seabirds, particularly black-browed albatrosses. Simulations were conducted for populations of black-browed albatrosses starting at various breeding colonies and running over a four month period: April to July, the core of the non-breeding season. Outside of this period we might expect movement decisions to also be governed by a need to return to breeding colonies, rather than purely by environmental factors. For each population, all migratory paths started at the corresponding breeding colony, and at each time step of the simulation the position of the simulated bird was updated according to the potential of surrounding lattice points and simple decision rules.

### The Environmental Potential Landscape

The potential landscape is defined by two factors: food resources and wind.

#### Attraction to Resources

**Figure 1:**
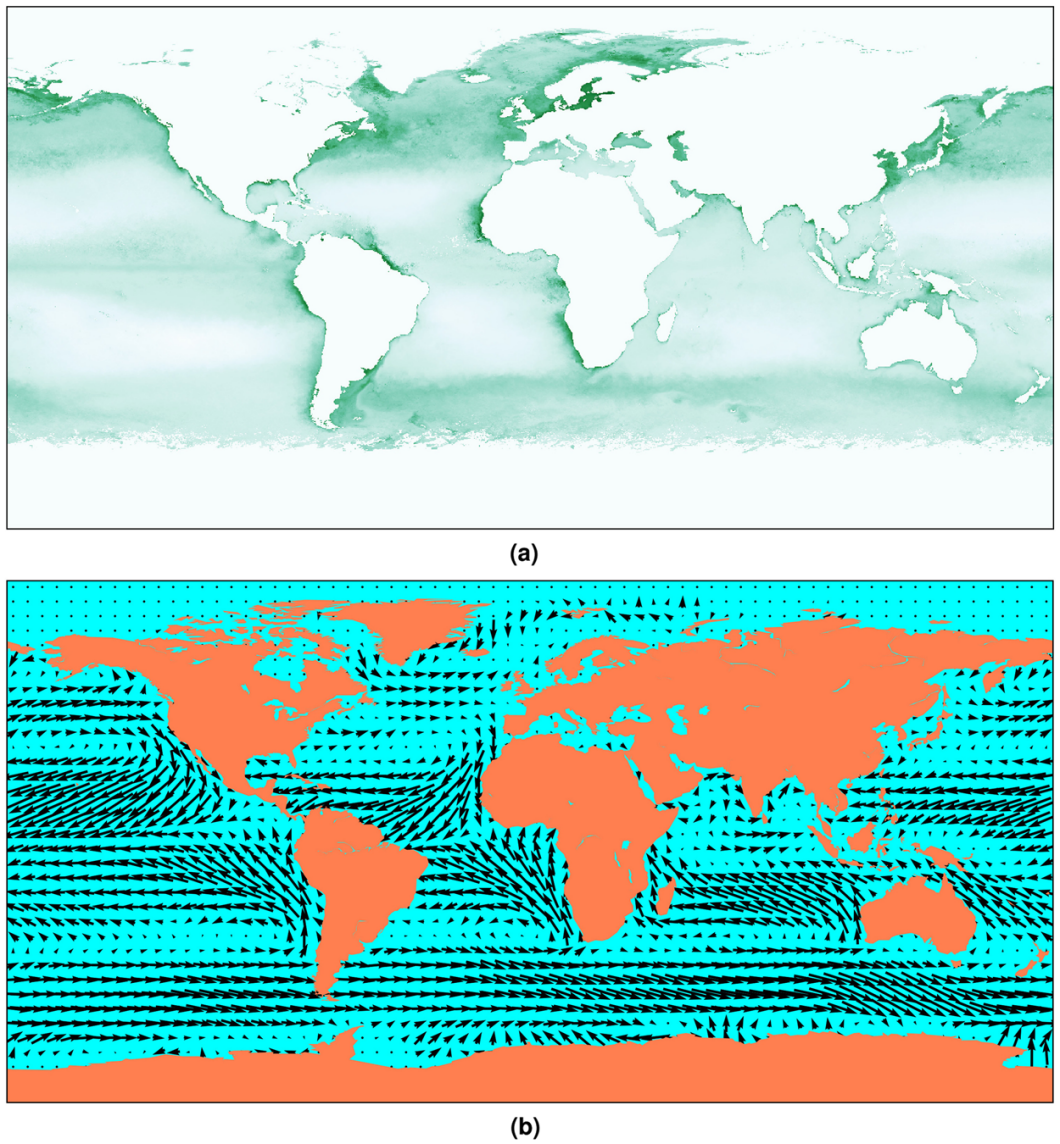
Environment data plots^41^ for April, averaged over all raw data between 2003 and 2010. (**a**) Chlorophyll concentration data^38^. Plotted as logarithm of actual value to highlight distribution patterns. Inland lakes excluded using ocean shapefile^40^. (**b**) Wind data^42^. Length of arrow indicates wind magnitude.

We assumed that birds have a full knowledge of the predictable geographical distribution of resources across the ocean and that two aspects of a food source play a role in its attractiveness: its density and its proximity^37^. We obtained remote sensing measurements of monthly chlorophyll a concentration from NASA^38, 39^, which we averaged for each month over the period 2003–2010 (the period over which both chlorophyll and wind data were readily available; see Fig. 1a for the averaged data from April). Such averaging ensured both that only the geographical patterns of chlorophyll a concentration that are predictable over the years are retained, and that the holes where cloud cover had prevented measurements from being taken did not create any gaps. The chlorophyll datasets also included contributions from inland lakes, such as Lake Victoria, which have unusually high chlorophyll concentrations. Since such regions are inaccessible to seabirds, we excluded them from calculations by overlaying a lattice of binary values describing the location of the oceans^40^, and excluding from calculations all chlorophyll a concentration values that did not fall in regions defined as ocean.

In order to translate resource density data to a potential landscape, we imagined each unit of chlorophyll a concentration as though it were a unit of charge or mass, producing a potential field that decays with distance, akin to an electrostatic or gravitational potential field. On a two dimensional lattice, this corresponds to a −1/*r* potential. Thus at any lattice point (*i*,*j*) the potential due to resources, *ϕ*_*ij*_ is given by Equation 1.

1 $${\varphi }_{ij}=-\sum _{k,l}\frac{{C}_{kl}}{{r}_{ij,kl}}$$

**Figure 2:**
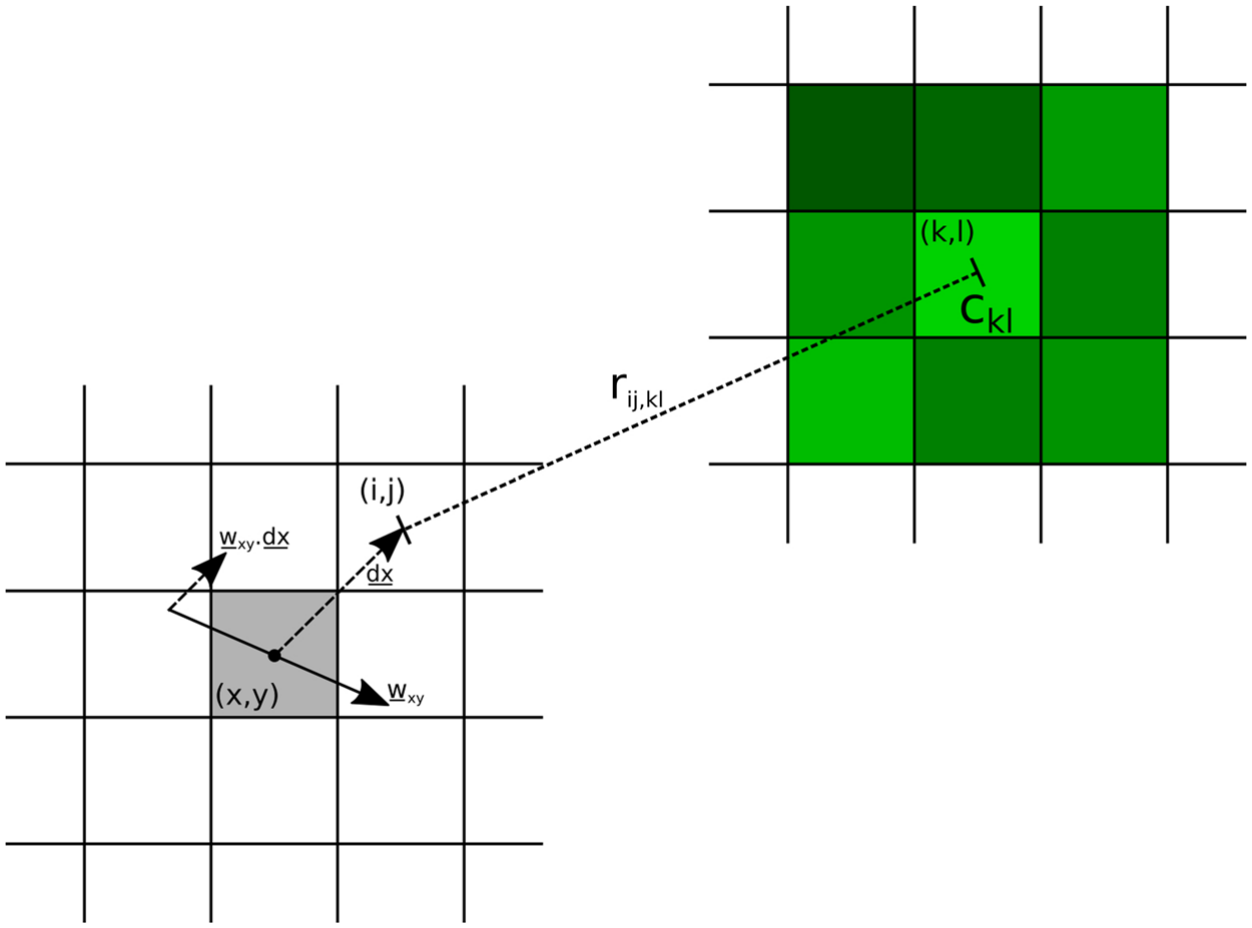
Diagram to demonstrate how potential at point (*i*, *j*), where (*i*, *j*) is a neighbour of the current bird location (*x*, *y*) (shaded grey), is calculated. Each lattice point (*k*, *l*) has a corresponding chlorophyll concentration, which produces a potential field proportional to the concentration that decays as 1/*r*_*ij*,*kl*_. The potential component due to resources at (*i*, *j*) is the sum of all such potential fields. The wind component of the potential at (*i*, *j*) is calculated from the dot product of wind vector ${\underline{w}}_{xy}$ at (*x*, *y*) with the displacement vector from (*x*, *y*) to (*i*, *j*).

The right hand side of Equation 1 is summed over all lattice points (*k*, *l*) where (*k*, *l*) ≠ (*i*, *j*). *C*_*kl*_ is the chlorophyll concentration at (*k*, *l*) and *r*_*ij*,*kl*_ is the distance from (*i*, *j*) to (*k*, *l*) [Fig. 2].

#### Wind

As for chlorophyll concentration data, we obtained wind data from NOAA^42, 43^, which we then averaged over the period 2003–2010 in order to fill gaps in the data. These data were in the form of one file per month, containing a mean surface wind speed across that month. The data were of the same resolution as the chlorophyll concentration data.

We used these data to construct a vector field covering the surface of the globe [Fig. 1]. Wind is a rotational (non-conservative) vector field, which renders it impossible to define a corresponding global scalar potential field^44^. Therefore, we calculate the wind component of the potential locally, as a tilt to the potential field around the current position. This tilt corresponds to a change in the potential at each of the lattice points that are available in the next timestep. It is defined such that moving downwind constitutes moving down a potential gradient, and is therefore preferred over moving upwind, which involves moving up a potential gradient. Moving perpendicular to the wind involves no change in potential. This potential is analogous to tailwind flow support^45^, and does not consider the possible effects of dynamic soaring in crosswinds^46^.

At lattice point (*x*, *y*), the wind vector obtained empirically from data is ${\underline{w}}_{xy}$. The force on a particle due to fluid drag, *F*, is proportional to the square of the velocity of the fluid relative to the particle: $F\,\alpha \,{|\underline{w}|}^{2}$ ^47^. Thus, considering that a change in potential is the work done against an opposing force, we hypothesised that the potential due to wind, *χ*_*ij*_, for any (*i*, *j*) where *i* = *x* ± 1 and *j* = *y* ± 1, is given by Equation 2.

2 $${\chi }_{ij}=-|{\underline{w}}_{xy}|({\underline{w}}_{xy}\cdot \underline{dx})$$

Note that we must be careful in the definition of $\underline{\hat{d}x}$, since it is tempting to take simply the normalised displacement in the lattice. However, we must remember that the wind data is a two dimensional vector defined on a spherical surface geometry. Therefore, we should not calculate its dot product with a vector that connects two lattice points in a flat Euclidean space. Although at small scales the surface of a sphere is approximately Euclidean, for a lattice point encompassing 0.25° of latitude and longitude we can be more accurate by defining $\underline{\hat{d}x}$ as a vector in the spherical surface geometry, connecting the points on the earth’s surface that correspond to the neighbouring lattice points in Euclidean space. The relative lengths of the zonal and meridional components of such a vector will vary across the globe.

Once the wind potential component for a lattice point (*i*, *j*), *χ*_*ij*_, had been calculated, it was summed with the chlorophyll component *ψ*_*ij*_ to obtain the total potential Ψ_*ij*_, as in Equation 3

3a $${{\rm{\Psi }}}_{ij}={\varphi }_{ij}+a{\chi }_{ij}$$

3b $${{\rm{\Psi }}}_{ij}=-\sum _{k,l}\frac{{C}_{kl}}{{r}_{ij,kl}}-a|{\underline{w}}_{xy}|({\underline{w}}_{xy}\cdot \underline{\hat{d}x})$$

Equation 3 introduces *a*, the first unknown parameter of the model.

### Updating Bird Position - Statistical Mechanics of Bird Movement

Once the potential landscape is defined from environmental data, we must consider the rules that determine how a bird moves within this landscape. For a bird at position (*x*, *y*), there are 8 possible positions that it could move into in the next time step, corresponding to the 8 lattice points immediately surrounding (*x*, *y*). Explicitly, this set of positions *S* is (*x*, *y* + 1), (*x*, *y* − 1),(*x* − 1, *y* − 1), (*x* − 1, *y*), (*x* − 1, *y* + 1), (*x* + 1, *y* − 1), (*x* + 1, *y*), (*x* + 1, *y* + 1). The bird is not permitted to remain at its current location. Each of these possible positions has an associated potential.

In order to determine which of these positions a bird will move into, we draw an analogy to statistical mechanics, specifically Maxwell-Boltzmann statistics. This theory posits that for a particle existing in a space with some set of possible states j with corresponding energy levels, the probability, *P*(*i*) of the particle occupying a state with energy *ε*_*i*_ is given by Equation 4.

4 $$P(i)=\frac{{e}^{-\beta {\varepsilon }_{i}}}{\sum _{j}{e}^{-\beta {\varepsilon }_{j}}}$$

In Equation 4, *β* is a constant inversely proportional to the temperature. ${e}^{-\beta {\varepsilon }_{i}}$ is the Boltzmann factor of energy level *ε*_*i*_. Thus the probability of occupying state *i* is the ratio of the Boltzmann factor of state *i* to the sum of the Boltzmann factors for all possible states^48^.

We can draw upon these ideas by treating the 8 possible positions *S* as 8 possible energy states of a particle, with the potential at each point defining the energy of that state. Thus the probability of moving from position (*x*, *y*) to position (*i*, *j*) where (*i*, *j*) ∈ *S*, is given by Equation 5, where indices *k* and *l* are summed over all states in *S*.

5 $$P(i,j)=\frac{{e}^{-\beta {{\rm{\Psi }}}_{ij}}}{\sum _{k,l}{e}^{-\beta {{\rm{\Psi }}}_{kl}}}$$

We can then apply a random number generator to select the next position for the bird according to these defined probabilities. Note that *β* or, equivalently, 1/*kT* is the second unknown parameter of the model, giving some measure of randomness or energy in a bird’s movements, analogous to an abstract temperature.

After the position has been updated, the clock is incremented by the time taken to travel this distance at the bird’s speed, which is itself a combination of a typical speed and a wind component. A typical airspeed of 60 *km*/*h* was used in all results presented here, and for this simple implementation dynamic soaring in crosswinds^46^ was not considered. This ends one iteration; iterations are repeated until the simulation time reaches the end of the specified run time (4 months). The chlorophyll attraction and wind experienced by a bird in the simulation are updated every month as time passes in the simulation.

Finally, note that we use the same map of ocean area that provided a mask for inland chlorophyll a concentration values^40^ to set the probability of moving to any lattice point of dry land to zero. Whilst is is possible for some seabirds, such as terns and skuas, to travel over land in some circumstances, this is less likely for albatrosses and for the purposes of simplicity we consider only movement over sea here.

### Implementation

To implement our model, we created a Python script^49^ that takes the unknown parameters as inputs and predicts the path of a single bird over a specified time period. The program imports monthly environmental data files and updates the potential landscape as time passes. To improve the efficiency of an otherwise slow program, all heavy calculations were transferred to subroutines written in Fortran, and integrated with the Python script using f2py^50^. This produces a dramatic improvement in run time and allows for vastly more efficient data collection, whilst retaining the ease of data manipulation from Python.

### Exploring the parameter space

Our model contains two unknown parameters: *a*, interpreted as the importance of the wind component relative to the attraction to resources in the definition of the environmental potential, and *kT*, interpreted as the degree of randomness in the movement decisions made by the simulated birds. For each of the eight breeding colonies, we ran simulations for 32 different parameter pairs: values of *a* as multiples of 0.001 from 0.001 to 0.008, and values of *kT* as multiples of 0.05 from 0.05 to 0.2. These values were chosen to demonstrate a full range of results. Above or below this range of *a* values, we found that wind and chlorophyll effects dominated, leading to no further variation in results; within this range of *a* values, we see a transition between dominance of either parameter, and find a point of balance. Above this range of *kT* randomness overwhelmed observed patterns. For each parameter pair at each location, we ran 16 individual simulations to obtain a representative sample of migration pathways. In lieu of full statistical analysis to determine the number of tests needed for significant results, and statistical comparison to tracking data, 16 individuals were found to provide a suitable range of results to explore and demonstrate patterns, within our computational limits. All results were mapped and collated into a diagram showing the full phase space for each location. These full phase spaces are shown in the Appendix. We visually and qualitatively compared the simulation results to published migration patterns based on tracking data for five breeding colonies (see Introduction for details).

## Results

**Figure 3:**
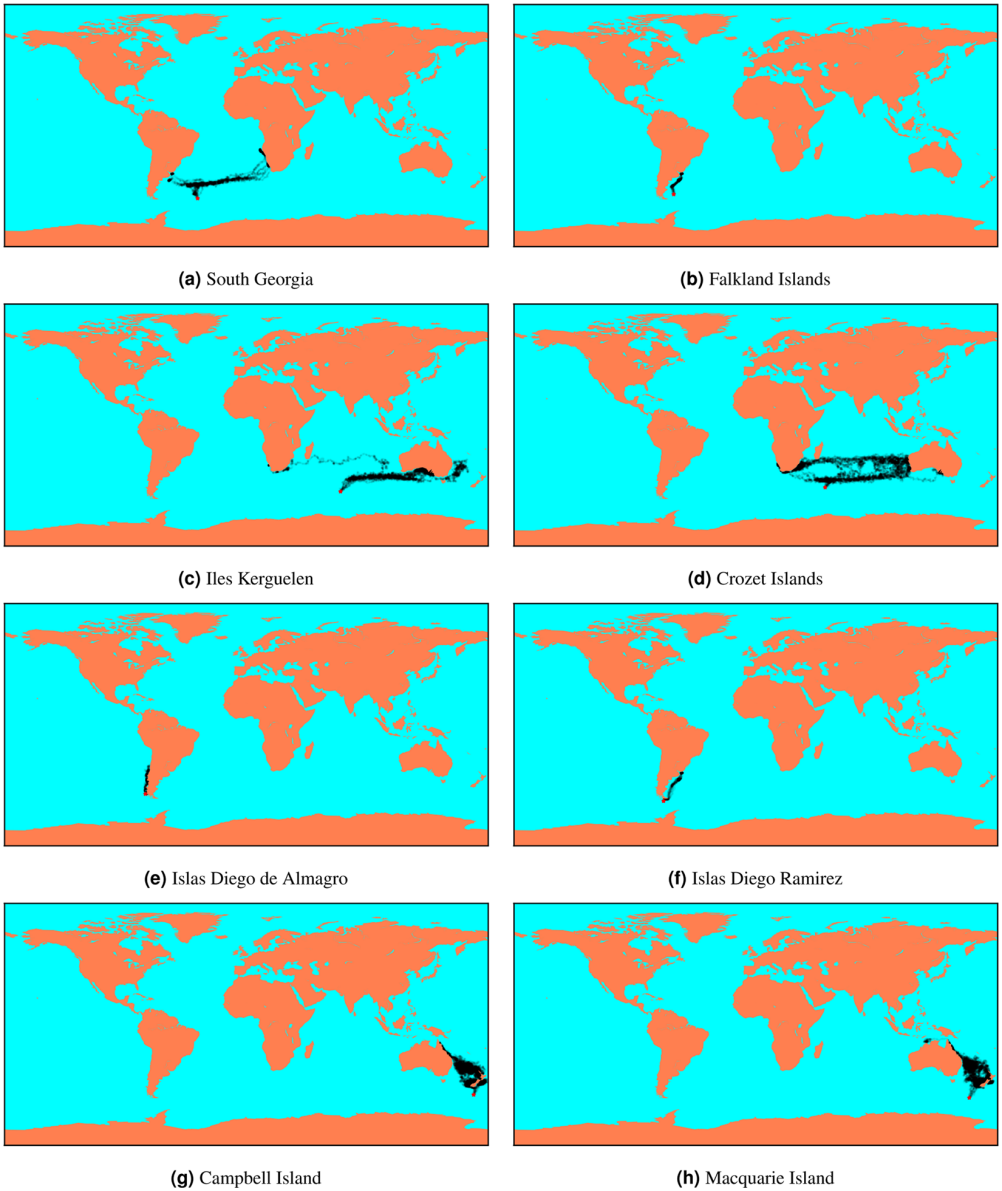
Results from 16 runs of the model at each location with a = 0.005 and kT = 0.1^41^.

We found that simulations with *a* = 0.005 and *kT* = 0.1 produced results that best matched the published movement patterns of black-browed albatrosses [Fig. 3]. For these parameter values, the model successfully predicted that the population breeding in South Georgia migrates to both the Patagonian Shelf and the Benguela Upwelling during the non-breeding season [Fig. 3a]. The model captured not only the main regions to which birds from this population have been observed to migrate, but also that a majority of birds will go to the south-west coast of Africa whilst a smaller fraction simultaneously migrates to the Patagonian Shelf. With these parameter values, the model also successfully predicted that the population breeding in Iles Kerguelen migrates to the southern coast of Australia, with a small fraction of the individuals finding its way to the coast of South Africa [Fig. 3c]. Furthermore, the model successfully predicted that birds from the Falkland Islands remain on the Patagonian Shelf [Fig. 3b] and that birds from Macquarie Island remain around New Zealand [Fig. 3h]. For the population breeding on Islas Diego Ramirez, the parameter values *a* = 0.005 and *kT* = 0.1 predicted that birds migrate to the Patagonian Shelf, whereas tracking data suggest that they instead migrate to the west coast of Chile^23^.

The full phase space of results showed that when the importance of wind was relatively low compared to attraction to resources (low values of *a*; e.g. *a* = 0.001), the model predicted that the birds from South Georgia move to the nearby Patagonian Shelf [Figure A.viii] and birds from Kerguelen migrate to the eastern coast of Africa, or more generally throughout the Indian Ocean when randomness is increased (high values of *kT*) [Figure A.iv]. Populations from the Falkland Islands, Islas Diego Ramirez and Macquarie Island showed no qualitative variation in distribution with changes in the value of *a* [Figure A.iii, A.vi, A.vii], except that some individuals from Islas Diego Ramirez migrate to the west coast of Chile when randomness is high. When the importance of wind is relatively high compared to attraction to resources (high values of *a*; e.g. *a* = 0.008), the model predicted that the population from South Georgia moves entirely to the coast of southern Africa and the Benguela Upwelling and the birds from Kerguelen migrate mainly to Australia and New Zealand, with some individuals still finding their way to south Africa when randomness is high.

By applying the best fit parameters for populations with known distributions (*a* = 0.005 and *kT* = 0.1; Fig. 3) we obtained interesting predictions for other populations that have been less well documented. The model predicted that birds from Islas Diego de Almagro, located north-west of Islas Diego Ramirez, move north along the west coast of Chile [Fig. 3e]. This pattern is similar to what is observed for the population from Islas Diego Ramirez but differs from the predictions produced by the model for Islas Diego Ramirez. This result was robust across the range of parameter values that we tested, only showing variation in northward extent for higher values of *kT* [Figure A.v]. Results for Campbell Island were similar to those from the nearby Macquarie Island, predicting that the population remains in the region of New Zealand and the Tasman Sea, with some individuals moving up the East coast of Australia towards Papua New Guinea [Fig. 3g]. These results again showed little variation across the range of parameter values tested [Figure A.i]. Finally, the population from the Crozet Islands was predicted to split roughly evenly between individuals migrating to Australia and those migrating to South Africa [Fig. 3d]. For low values of *a*, birds only go to southern Africa, whereas for high values of *a* birds mainly go to Australia [Figure A.ii]. Notably, for all parameter pairs, the results for the Crozet Islands showed more individuals migrating to South Africa and fewer to Australia than for the corresponding results from nearby Iles Kerguelen, with the population roughly equally split between the two for the best-fit parameter values (*a* = 0.005 and *kT* = 0.1; Fig. 3).

## Discussion

The mechanistic model that we have developed yielded very promising results. It was able to qualitatively capture the non-breeding migration patterns of black-browed albatross populations from various breeding colonies across the southern Hemisphere using simple decision rules based on a minimal amount of environmental data and requiring only two unknown parameters. In addition, the best combination of parameter values was conserved across populations – all the best matches occur around a = 0.005 and kT = 0.1 [Fig. 3]. This strongly suggests that our model captures the fundamental forces driving migration in black-browed albatrosses.

South Georgia and Iles Kerguelen provided interesting test for exploring the behaviour of the model. Both are located relatively far from continental land masses and high productivity areas, and both populations split into a majority of individuals migrating eastward while some individuals migrate westwards^33, 34, 36^. For both populations, the model behaved in a similar way. The most accurate predictions were obtained for parameter values around a = 0.005 and kT = 0.1. When the importance of wind was relatively low compared to attraction to resources (low values of *a*) the birds tended to mostly migrate westwards whereas with a higher wind component birds tended to migrate eastward. This effect is due to predominantly strong easterly winds in the southern Ocean [Fig. 1b]. The fact that qualitatively accurate predictions for these two populations were obtained for the same restricted combination of parameter values is very encouraging.

We also used the model to make predictions for the non-breeding migration of black-browed albatrosses from the Crozet Islands, another fairly remote breeding colony not far from Iles Kerguelen. The model predicted that this population should follow a similar migration pattern to the one from Iles Kerguelen, but with a higher proportion of individuals migrating westwards to South Africa and fewer migrating eastwards to Australia. This could be an interesting prediction to test when data becomes available for this population.

Black-browed albatrosses breeding on the Falkland Islands, the largest breeding colony for this species^30^, were accurately predicted to migrate to the nearby Patagonian Shelf. Birds from Macquarie Island were also accurately predicted to remain around New Zealand and the Tasman Sea. In contrast to birds from South Georgia and Iles Kerguelen, these results show little variation across *a* or *kT* [Fig. 3b,h], which renders the results less informative but still remains a successful set of predictions from the model.

The only major discrepancy between the predictions of our model and experimental observations of black-browed albatrosses was for Islas Diego Ramirez, located at the southern tip of Chile. The model predicted that this population should migrate to the Patagonian Shelf, attracted by the productive seas and driven by the strong easterly winds across Cape Horn [Fig. 3f], whereas they have actually been observed to migrate along the west coast of Chile^23^. This discrepancy could potentially be explained by competition between the population from Islas Diego Ramirez and the large population from the Falkland Islands. Both of these populations are predicted to migrate to the Patagonian Shelf [Fig. 3] and since the population from the Falkland Islands is nearer to the Patagonian Shelf and greater in numbers, it is possible that the Falkland Islands population excludes the Islas Diego Ramirez population from this region, forcing them to migrate elsewhere in order to find sufficient food. This effect has previously been suggested for foraging activity during the breeding season^7, 51^. The model predictions for Islas Diego Ramirez contrasted to the predictions for nearby Islas Diego de Almagro, located not far to the north of Islas Diego Ramirez. This population was indeed predicted to migrate along the Chilean coast [Fig. 3e] as they appear to avoid the strong winds near Cape Horn. It is therefore possible that very little competitive exclusion from the overcrowded Patagonian Shelf would be required for the Islas Diego Ramirez population to follow similar pathways to predicted pathways for the population from Islas Diego de Almagro.

Competition for access to resources (either intra- or inter-specific) has been suggested as a factor affecting the geographical distribution of seabird populations during their non-breeding season. Birds tend to segregate spatially to avoid overcrowding, which would cause lower food availability per individual^1, 52^. As mentioned above, competition could be a major factor in explaining why our model fails to correctly predict the distribution of birds from Islas Diego Ramirez. In addition, competition could explain why a small number of individuals from South Georgia travel past South Africa to Australia, a feature that is not currently predicted by the model [Fig. 3a]. Since a large number of individuals are already exploiting the productive areas of the Benguela Upwelling off southern Africa (South Georgia being the second largest breeding colony for this species^30^), such competition at the Benguela Upwelling might force some individuals to migrate further in order to access less crowded areas. It would therefore be very interesting to incorporate competition into the model and investigate its impact on the predicted distributions, and its significance relative to the other processes. Competition could either be included directly into the potential landscape of our model with a repulsive potential between individuals, or by modifying the attractive potential associated with resource density as the number of birds in the vicinity of the resources increases. Competition could also be incorporated using a game theoretic approach^18^, in which a variety of movement behaviours could co-exist in a population, and evolutionary programming^18^ to find the optimal distribution of individuals of a population given intra-specific competition.

Another ecological process that has been ignored in this study, but which could be important for explaining some failures of the model as well as for making predictions for other species, is niche tracking. Each species has an ecological niche - a set of environmental conditions, such as temperature and habitat, to which it is best adapted - and this is likely to play a role in determining its geographical distribution. Black-browed albatrosses, for example, prefer cool waters over warm waters^7^, which might help to explain why our model predicted that some birds from Macquarie Island migrate up the east coast of Australia towards Papua New Guinea, a feature that is not observed in tracking data (although data for this population come from a very small number of individuals). The thermal niche of a species could be incorporated into the model by, for example, introducing a potential component proportional to sea surface temperature data, thus increasing the potential of warmer areas and reducing the probability that a bird will move into these areas.

In addition to expansions to the model itself, it would be interesting for future studies to conduct full quantitative analysis of the fit of this mechanistic model to tracking and distribution data, since we have relied here only on a qualitative comparison of simulation results to broadly known and previously published population distributions. Precise, quantitative parameter fitting, for example using the Earth mover’s distance method^53^ to compare tracked distributions with simulated results, would allow precise identification of the best-fit combination of parameter values for all locations. Performing this fitting for different species at various breeding locations would allow us to infer the relative importance of different mechanisms in determining the non-breeding migration of different species of seabirds. For example, our model could potentially explain differences in migration patterns for different species breeding on the same island, such as the white-chinned petrels and black-browed albatrosses that share breeding grounds on South Georgia but migrate to different non-breeding areas – the former migrating around Patagonia^54^ while the latter mainly migrates to the Benguela Upwelling off southern Africa^36^. This would indicate that the best-fit value of parameter *a* is lower for white-chinned petrels than for black-browed albatrosses. Since the value of a indicates the importance of wind to a given migration pathway, this would perhaps indicate that wind is a more important factor in the distribution of black-browed albatrosses than white-chinned petrels. After obtaining a different set of best-fit parameter values for each species, it would be very interesting to test if these values are consistent with the different biology of the species. For example, is the importance of wind consistent with the morphology and behaviour of the species? We might expect larger birds that rely more on soaring for locomotion or that have larger wingspans to fit results with higher wind components, as is seen for the comparison between black-browed albatrosses and the smaller white-chinned petrels. If this is the case we could interpret parameter *a* as related to the morphology of a species. Thus our model could be used to uncover a mechanistic explanation of why different species have different non-breeding migration patterns.

Alternatively, for populations within the same species, we can consider parameter *a* to be a measure of how hard the population is willing to work against the wind in order to access resources. For example, given two colonies of the same species whose migration patterns differ in the best-fit value of parameter *a*, we can suppose that the population whose migration pattern fits results from the lower value of *a* has worked harder against the wind to achieve its observed distribution. The reason for such an observed difference may be due to parameters not considered in the model, such as competition and niche-tracking, as discussed above.

Our model is intended to be widely applicable in its ability to model migratory movement patterns. Here, we explore a minimal version of the model that only includes two environmental factors and one biologically relevant parameter (in addition to a randomness parameter) by investigating its ability to capture the non-breeding migration patterns of a seabird species. It will be interesting to apply this model to other bird species in future, potentially including other environmental potential components. For example, modelling migratory land birds might require including breeding and non-breeding grounds, which tend to be fixed for each individual, as gravitational attractors in the environmental potential landscape. Remote sensing data of vegetation density could be used as a proxy for resource availability on land. In addition, our model might also be applied to other migratory organisms, such as walking organisms like grazers on African savannahs that are attracted by vegetation quality and water availability^55^, or swimming organisms like marine mammals and fish that are attracted by ocean primary production and affected by currents^4^.

In conclusion, we have developed a minimal, two-parameter model of migratory movement patterns based on the definition of an environmental potential landscape from environmental data. Exploring the capacity of this model for predicting seabird migration, we found an excellent qualitative match between simulation results and published tracking data from black-browed albatrosses (*Thalassarche melanophris*), with the best combination of parameter values conserved across multiple geographically separate populations. Such results show the potential for a physics-inspired mechanistic approach to ecological modelling, providing causal understanding of what determines observed patterns. Our model can thus improve our understanding of the relative importance of various factors driving the migration patterns of birds and other animals. With migratory animals forming a particularly threatened group of organisms^56^ (the black-browed albatross, for example, is classified by the IUCN as Endangered^57^), mechanistic models such as the one presented here will be important for making predictions under scenarios of environmental change, which will be needed for the future conservation of these species.

## Electronic supplementary material

supplementary information
