# Supplementary material for: A Physics-Inspired Mechanistic Model of Migratory Movement Patterns in Birds
Source: Sci Rep. 2017 Aug 29;7:9870. doi: 10.1038/s41598-017-09270-6 (PMC5574917; doi:10.1038/s41598-017-09270-6)
Supplement: Supplementary file 1 — supplementary information [file 41598_2017_9270_MOESM1_ESM.pdf]

A Physics-Inspired Mechanistic Model of Migratory Movement Patterns in Birds

Christopher Revell[1,*] and Marius Somveille[2]

[1]Cavendish Laboratory, Department of Physics, University of Cambridge
[2]Edward Grey Institute, Department of Zoology, University of Oxford
[*]cr395@cam.ac.uk

Appendix: Full Parameter Space Results

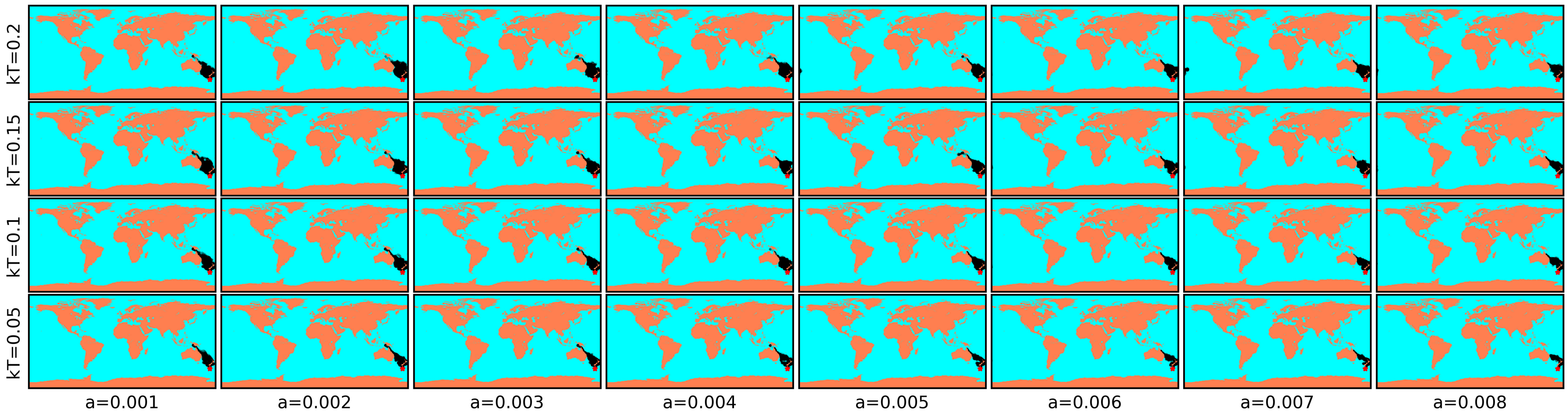

Figure A.i. Campbell Island Parameter Space[41]

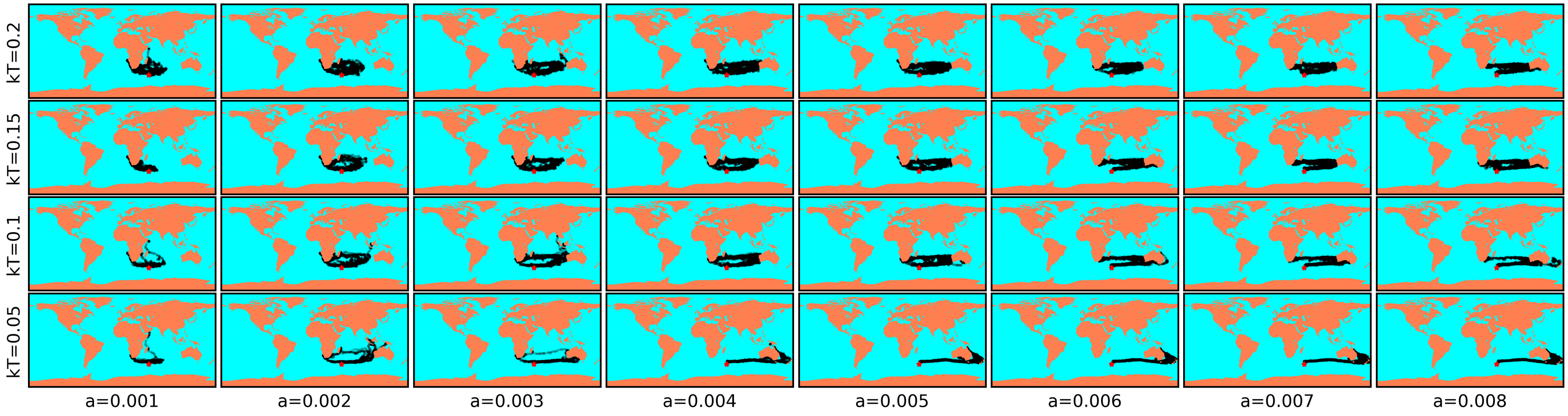

Figure A.ii. Crozet Islands Parameter Space[41]

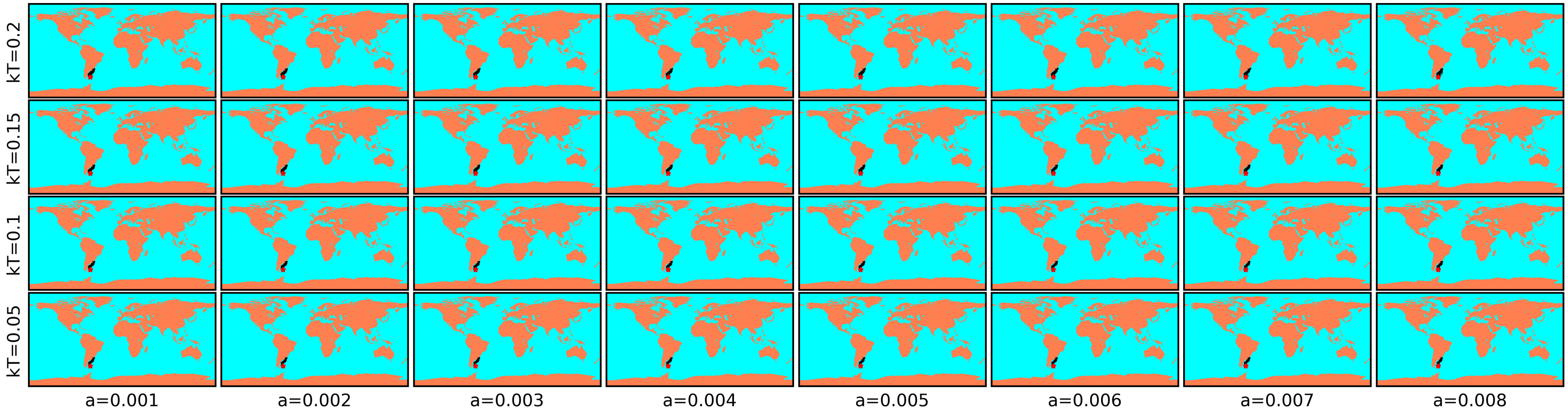

Figure A.iii. Falkland Islands Parameter Space[41]

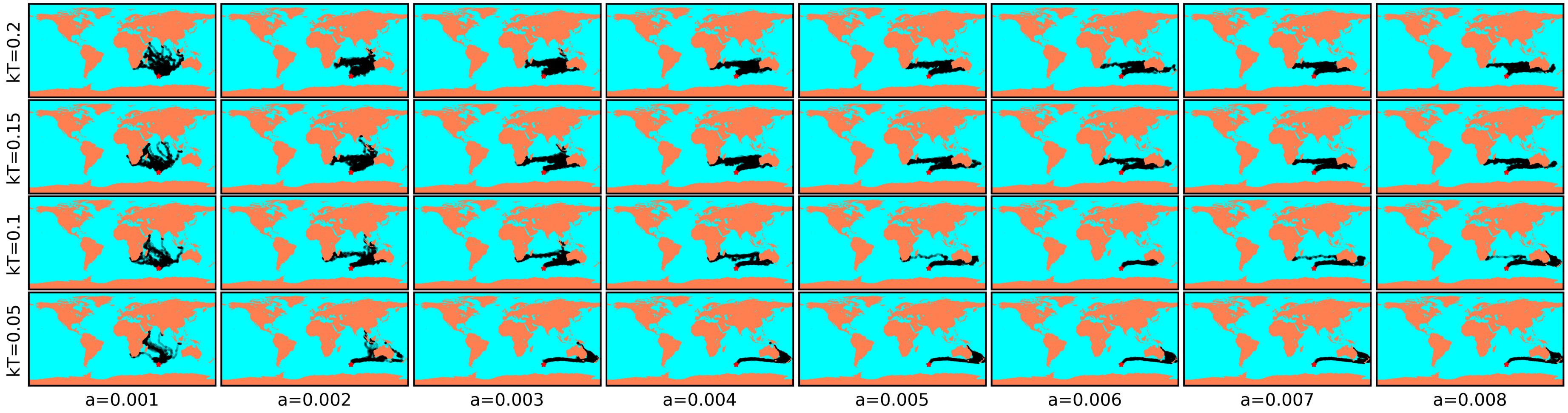

Figure A.iv. Iles Kerguelen Parameter Space[41]

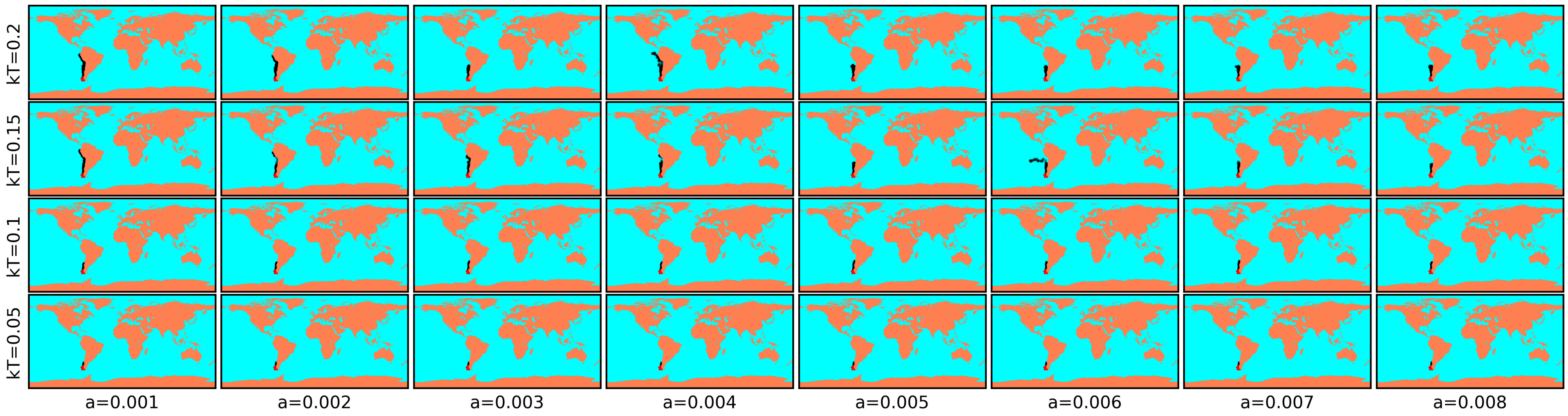

Figure A.v. Islas Diego de Almagro Parameter Space[41]

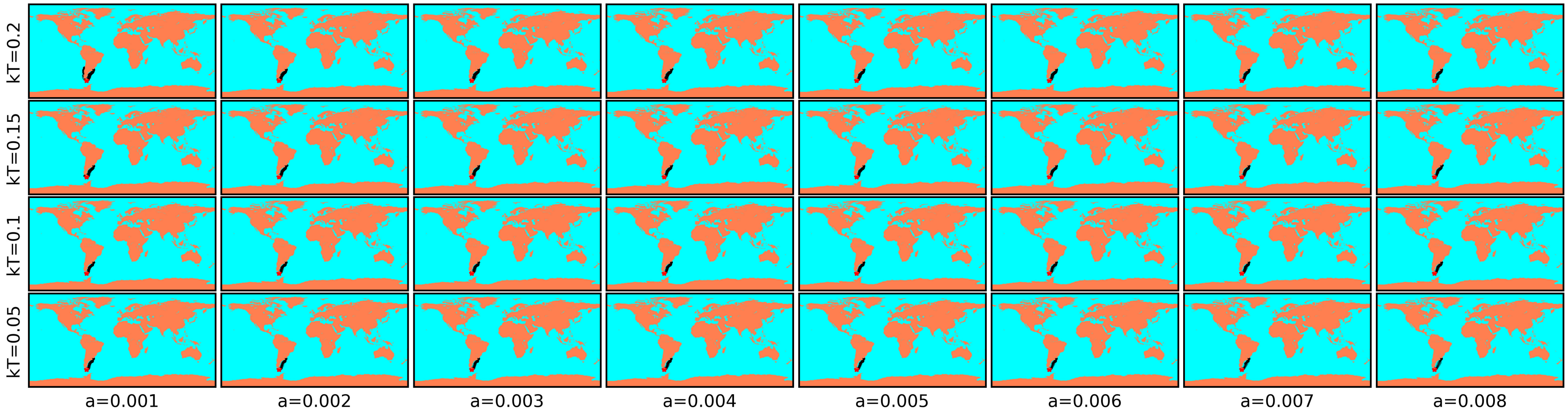

Figure A.vi. Islas Diego Ramirez Parameter Space[41]

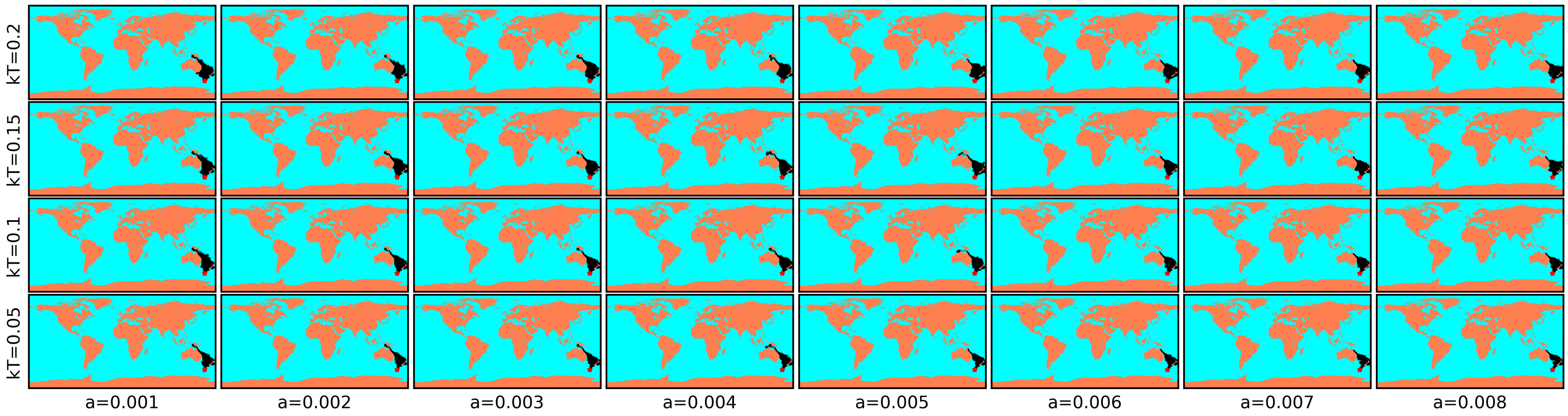

Figure A.vii. Macquarie Island Parameter Space[41]

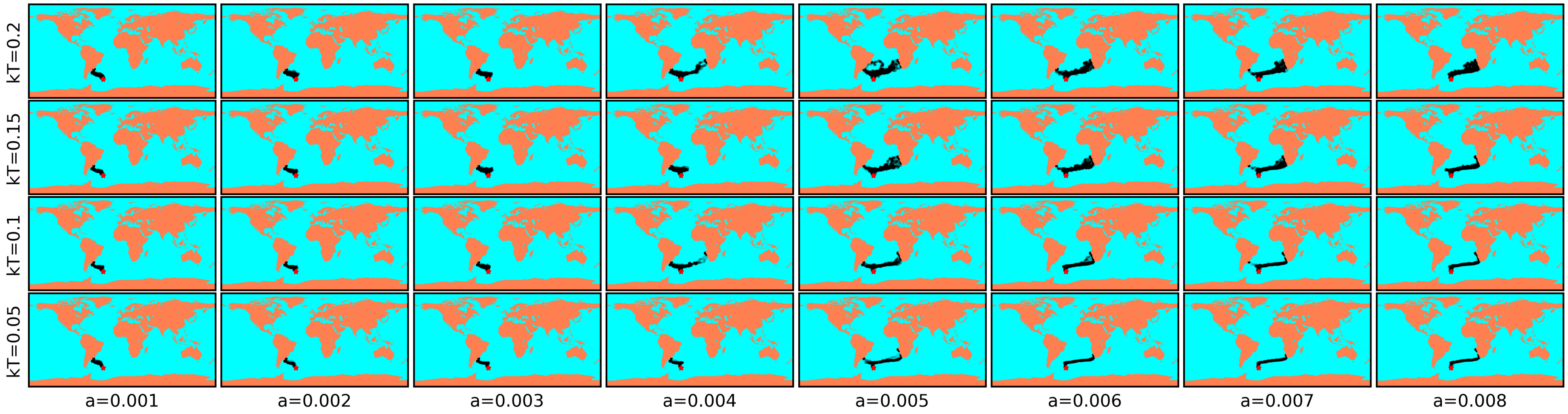

Figure A.viii. South Georgia Parameter Space[41]
